# Gut Microbiome of Two Rodent Species (*Niviventer confucianus* and *Apodemus agrarius*) from Two Regions Exhibit Different Structures and Assembly Mechanisms

**DOI:** 10.3390/ani15213187

**Published:** 2025-11-01

**Authors:** Haotian Li, Qian Gao, Jiawen Han, Qiuyue Song, Fangheng Yan, Yunzhao Xu, Chuansheng Zhang, Xin Wang, Yuchun Li

**Affiliations:** 1 College of Agriculture and Biology, Liaocheng University, Liaocheng 252059, China; lihaotian@lcu.edu.cn (H.L.); gao041231@126.com (Q.G.); 17663501303@163.com (J.H.); m19954219214@163.com (Q.S.); 2023404098@stu.lcu.edu.cn (F.Y.); x18653667611@163.com (Y.X.); 15589143830@163.com (C.Z.); 17563567862@163.com (X.W.); 2 Marine College, Shandong University (Weihai), Weihai 264209, China

**Keywords:** gut microbiome, co-occurrence network, assembly mechanisms, *Niviventer confucianus*, *Apodemus agrarius*

## Abstract

The structure and assembly mechanism of wild animal gut microbiota represent persistent research hotspots. Among, the impact of geographic factors on the bacterial co-occurrence network characteristics and assembly mechanism of the gut microbiome remains unclear. Therefore, this study analyzed the gut microbiome of *Niviventer confucianus* and *Apodemus agrarius* from Anhui and Hubei provinces. The same alpha diversity pattern was found in the gut microbiome of species from the same region. The gut microbiome of the two rodent species in Anhui region exhibited “small world” characteristics, such as nodes with more local connections to allow interaction information (such as metabolites) to rapidly spread throughout the entire microbial community. In addition, dispersal limitations and heterogeneous selection accounted for higher proportions of the gut microbiome in the rodents from the Anhui and Hubei regions, respectively. The higher proportion of heterogeneous selection may exacerbate selection pressure in the Hubei region. Multiple regression on distance matrices analysis revealed that geographic region exerted a limited but significant influence (0 < R^2^ < 0.2, * *p* < 0.05) on the gut microbiome, surpassing the effects of host phylogeny, gender, and weight. Nevertheless, the roles of regional factors—such as environmental microbes, pollutants, and diet—remain unexamined, and their potential as key drivers of microbiota variation in these rodents warrants further investigation.

## Simple Summary

The influence of different regions on the structure and assembly mechanisms of the animal gut microbiome has long been of interest to scientists. This study investigated the gut microbiome of *Niviventer confucianus* and *Apodemus agrarius* collected from Anhui and Hubei provinces. We aimed to characterize the bacterial co-occurrence networks and elucidate the assembly mechanisms of the gut microbiota in conspecific hosts across different geographical regions. We found that the geographic region shaped the gut microbiome of conspecific rodents by altering the alpha diversity, co-occurrence networks, and assembly processes. However, further analysis is needed to determine which factor of the two geographical regions is the main factor affecting the gut microbiota of the two rodent species.

## 1. Introduction

The gut microbiome has been proven to be a complex ecosystem containing diverse and complex microbial communities that energize host life activities [1], supplement the digestive capacity [2], and maintain gut homeostatic balance [3]. In addition, the gut microbiome has been found to play an important role in enhancing animal endocrine [4], perception [5], memory functions [6], pathogen defense [7], and toxin metabolism [8]. Increasing evidence suggests that various internal and external factors, such as host genotype, diet, social network, and habitat environment, affect the composition and function of the gut microbiota in host animals [9,10,11,12,13].

Studies have also shown that geographic region is one of the most important factors influencing the gut microbiome composition of the same species. For example, 16% of the variation in the gut microbiome of wild house mice in Western Europe can be attributed to the geographical region [14]. Wang found significant differences in the alpha diversity of the rodent gut microbiome from different geographical locations (diet and environments) but similar gut microbiome compositions in different rodents from the same location [15]. Moreover, geographic region (shared microbial pool) was significantly correlated with the alpha diversity of the gut microbiome in rodents, whereas host genotype had a minimal effect on the abundance of the microbiota [14,16]. However, in other rodent species, host genetic identity has been shown to be a better predictor of microbiome structure and diversity [17,18]. Previous studies have not investigated the co-occurrence network characteristics and assembly mechanisms of the gut microbiomes of the same species from different regions. In different geographic regions, how do the co-occurrence network characteristics and assembly mechanisms of the gut microbiome differ in the same rodent? An open question is whether the established convergence in alpha diversity of gut microbiome in sympatric rodents also applies to their co-occurrence network characteristics and assembly mechanisms. Based on the above studies, we hypothesized that while geographic regions drive divergence in co-occurrence network characteristics and assembly mechanisms of gut microbiome within a species, sympatric rodent species will display convergence in these characteristics.

Our field survey identified *Apodemus agrarius* and *Niviventer confucianus* as the dominant rodent species in Hubei and Anhui provinces. Building upon previous extensive studies on the gut microbiota of *A. agrarius* and other rodents across different geographical regions [15,16], we collected samples of *A. agrarius* and *N. confucianus* from two distinct habitats—the mountainous areas of Anhui and the farmland of Hubei. To address our proposed scientific question, we characterized the gut microbiome structure, co-occurrence network characteristics, and assembly mechanisms of the two rodent species from two regions using 16S rRNA sequencing, bacterial co-occurrence network, and null model analyses.

## 2. Materials and Methods

### 2.1. Sample Collection and CYTB Gene Sequencing

The rodent feces used in this research were collected on 24 December 2019, in Anhui Province (Anhui Dabie Mountain Tourist Scenic Area, Lu’an City; average temperature in December, 3 °C; altitude, 234 m) and 18 January 2020, in Hubei Province (Jiufogang Village, Taohuashan Town, Shishou City; average temperature in January, 2 °C; altitude, 36 m). The distance between the two locations is approximately 500 km. We used Sherman live traps (7.62 × 8.89 × 22.86 cm; H. B. Sherman Traps, Inc., Tallahassee, FL, USA) to capture *Niviventer confucianus* and *Apodemus agrarius*, and the feces of each species was collected separately and used for sequencing. After collecting the feces samples, the rodents were immediately released into the wild. In total, 12 *N. confucianus* (ANC: 6 specimens of *N. confucianus* from Anhui, HNC: 6 specimens of *N. confucianus* from Hubei) and 10 *A. agrarius* (APA: 5 specimens of *A. agrarius* from Anhui, HPA: 5 specimens of *A. agrarius* from Hubei) were sampled. Detailed information on the rodent feces is shown in Table S1. Fecal samples were stored in sterile tubes and maintained at −20 °C during transfer. Before sequencing, all samples were stored in a −80 °C freezer at Liaocheng University.

Total DNA was extracted from the feces using the QIAamp DNA Stool Mini Kit (QIAGEN, Hamburg, Germany), following the manufacturer’s protocol. We amplified and sequenced the complete mitochondrial protein-coding gene cytochrome b (*CYTB*, 1140 bp) obtained from 22 individuals (12 from *N. confucianus* and 10 from *A. agrarius*) following the protocol described by Li et al. [19]. The sequences were manually edited in BioEdit v.7.2.5 [20] and aligned using MEGA 11 [21]. The *CYTB* gene sequences were subjected to BLAST+ v.2.17.0 analysis against the NCBI (National Center for Biotechnology Information) database, and species identification was determined using the percent identity threshold (PI) and query coverage (QC) methods. Phylogenetic status was determined using the maximum likelihood method (ML) based on the *CYTB* gene sequences in MEGA 11, with the GTR + G + I model. The bootstraps were obtained using a rapid bootstrapping algorithm with 1000 replicates.

### 2.2. 16S rRNA Gene Sequencing, Sequence Processing, and Bioinformatics Analysis

We used universal primers (338F, 5′-ACTCCTACGGGAGGCAGCA-3′; 806R, 5′- GGACTACHVGGGTWTCTAAT-3′) to amplify the V3–V4 hypervariable regions of bacterial 16S rRNA genes. The PCR amplification was performed in a 25 μL reaction mixture containing 5 μL reaction buffer (5×), 5 μL GC buffer (5×), 2 μL dNTP (2.5 mM), 1 μL forward primer (10 μM), 1 μL reverse primer (10 μM), 2 μL DNA template, 8.75 μL ddH_2_O, and 0.25 μL DNA Polymerase. The PCR conditions were as follows: initial denaturation at 98 °C for 2 min; 30 cycles of denaturation at 98 °C for 15 s, annealing at 65 °C for 30 s, and elongation at 72 °C for 30 s; and a final elongation at 72 °C for 5 min. DNA libraries were prepared from the PCR products using a TruSeq^®^ DNA PCR-Free Sample Preparation Kit (Illumina, San Diego, CA, USA) according to the manufacturer’s protocol. DNA libraries were sequenced on an Illumina NovaSeq PE250 platform (Illumina, USA).

Raw sequences were analyzed using EasyAmplicon software v.1.18.1 [22], with the Vsearch plugin used to merge paired-end reads of raw data, cut primers, and remove redundancies to generate a non-redundant sequence catalog. The unoise3 algorithm was used to denoise the sequences and cluster them into amplicon sequence variants (ASVs). The ASVs were annotated using the SILVA database v.138 with a threshold of 0.8–1 [23]. The Richness, Shannon, and Good’s coverage indices (alpha diversity) and Bray–Curtis distance (beta diversity) were calculated using the QIIME package v.191 [24]. The Tutools platform (http://cloudtutu.com.cn/) was used to generate rarefaction curves and species accumulation boxplots and perform principal coordinate analysis (PCoA), non-metric multidimensional scaling (NMDS), and analysis of similarities (ANOSIM). STAMP software v.2.1.3 was used to detect bacteria with significant differences between regions [25]. A Spearman correlation coefficient (|r|) threshold > 0.7 and *p* < 0.001 were calculated after filtering operational taxonomic units with a mean relative abundance of less than 0.01% and accounting for less than 1/2 of the total sample size using R software v.4.5.1 (packages: WGCNA, psych, reshape2, and igraph), and network visualization was performed using Gephi v.0.10.1. The null model was also calculated and plotted using R software v.4.5.1 (packages: picante, parallel, ape, dplyr, and ggplot2).

### 2.3. Multiple Regression on (Dis)Similarity Matrices (MRMs)

The multiple regression on (dis)similarity matrices (MRMs) method was used to investigate the associations of host phylogeny (phylogenetic branch length), geographic location (latitude and longitude), gender (male and female were coded from 1 and 2, respectively), and weight with gut bacterial community α-diversity (i.e., Shannon index) and β-diversity distance (Bray–Curtis distances). Detailed information on phylogenetic branch length (total branch length), geographic location, gender, temperature, altitude, and weight for the two rodent species is shown in Table S1. The correlation coefficient (R^2^) and *p*-value were determined employing the MuMIn and ecodist packages in R software, with datasets were sampled 1000 times.

## 3. Results

### 3.1. Species Identification

In this study, the two rodent species were preliminarily identified based on their morphological characteristics. Then, we successfully amplified the complete 1140 bp mitochondrial *CYTB* sequences for 21 individuals except S4889. The BLAST search revealed rodent information using the *CYTB* sequence. All QC and PI values exceeded 99%, thus providing strong support for the reliability of the species identifications (Table S2). Although sequence data for S4489 were not obtained, the molecular results from all other specimens consistently supported the preliminary morphological identifications. Based on this congruence, individual S4889 was classified as *A. agrarius*. The *CYTB* phylogenetic tree displays the genetic relationships between 21 individuals in Supplementary Materials (Figure S1).

### 3.2. Data Statistics

**Figure 1:**
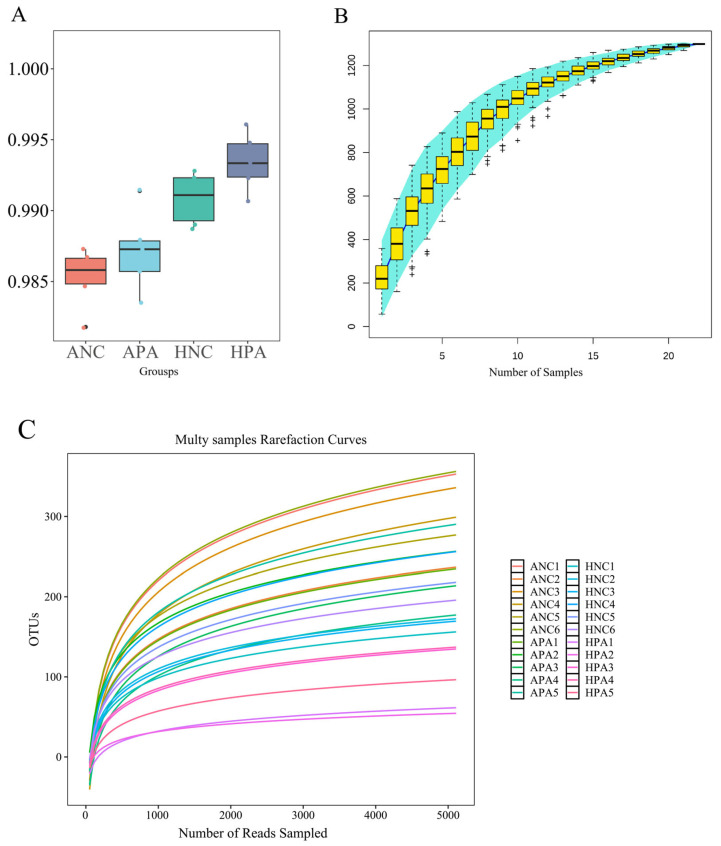
Boxplot of Goods coverages index between four groups (**A**), species accumulation boxplot (**B**), and rarefaction curves (**C**). ANC: *N. confucianus* in Anhui Province; HNC: *N. confucianus* in Hubei Province; APA: *A. agrarius* in Anhui Province; HPA: *A. agrarius* in Hubei Province. The species accumulation boxplot and multiple rarefaction curves reached a plateau, implying that the sequenced samples were adequate for subsequent analysis.

After data quality control, 941,604 effective tags were obtained from 22 samples, for an average of 42,800. 18 effective tags per sample. The effective tags were classified into 1864 ASVs after the unoise3 algorithm analysis. The lower quartiles of the Good coverage index box plot (Figure 1A) were all above 90%, indicating that the bacterial communities in the samples effectively represented the bacteria in the guts of *N. confucianus* and *A. agrarius*. The species accumulation boxplot (Figure 1B) and 22 rarefaction curves (Figure 1C) were close to a plateau, indicating that the sample size and sequencing depth used in this study were reasonable for subsequent analyses.

### 3.3. Composition and Differences in the Gut Microbiome

**Figure 2:**
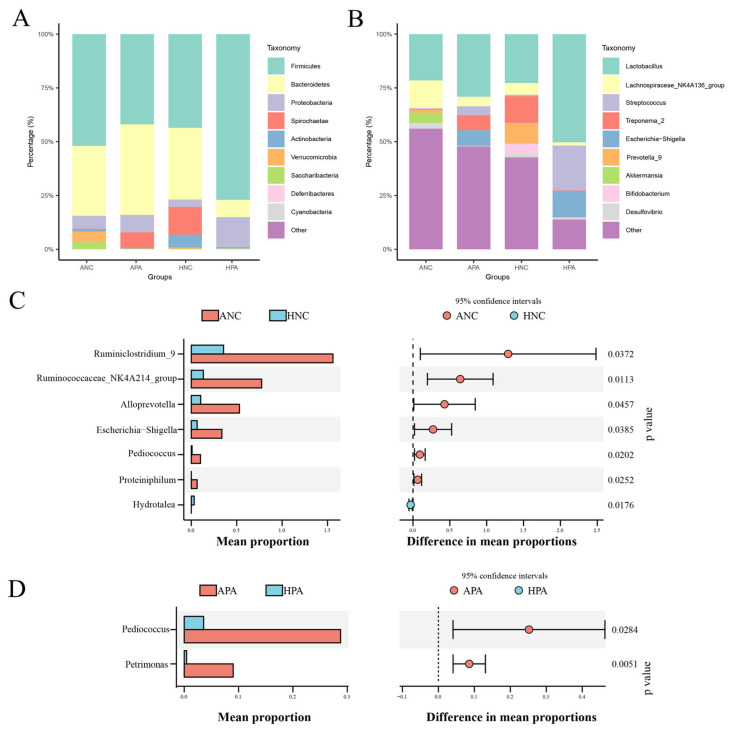
Gut microbiome composition of the two rodent species from two regions. Cumulative relative abundance plot of the top 10 bacteria at the phylum (**A**) and genus (**B**) levels. At the genus level, STAMP analysis between same species from two regions ((**C**), ANC vs. HNC; (**D**), APA vs. HPA). ANC: *N. confucianus* in Anhui Province; HNC: *N. confucianus* in Hubei Province; APA: *A. agrarius* in Anhui Province; HPA: *A. agrarius* in Hubei Province.

At the phylum level (Figure 2A), Firmicutes (ANC, 29.50–69.80%; APA, 28.10–77.10%; HPA, 27.30–96.80%), Bacteroidetes (ANC, 26.70–49.5%; APA, 14.10–70.80%; HPA, 0.84–16.00%), and Proteobacteria (ANC, 2.52–15.10%; APA, 0.61–35.20%; HPA, 0.20–61.00%) were the main phyla in ANC, APA, and HPA, while Firmicutes (11.90–74.00%), Bacteroidetes (12.40% ± 59.80%), and Spirochaetae (0.039–39.20%) the main phyla in HNC. At the genus level (Figure 2B), *Lachnospiraceae NK4A136 group* (3.54–30.00%) was the most abundant in ANC, while *Lactobacillus* was the most abundant in APA (2.62–29.60%), HPA (12.80–86.90%), and HNC (2.41–66.9%).

At the genus level, differential bacteria in the same rodents from Anhui and Hubei provinces were identified by STAMP analysis (ANC vs. HNC; APA vs. HPA; *p* < 0.05). In Anhui Province, STAMP identified differences in the relative abundance of six genera (*Ruminiclostridium 9*, *Ruminococcaceae NK4A214 group*, *Alloprevotella*, *Escherichia-Shigella*, *Pediococcus*, and *Proteiniphilum*) and two genera (*Pediococcus* and *Petrimonas*) in ANC and APA, respectively (Figure 2C,D). However, in Hubei, only *Hydrotalea* was significantly enriched in *N. confucianus* (Figure 2C).

### 3.4. Alpha and Beta Analyses

**Figure 3:**
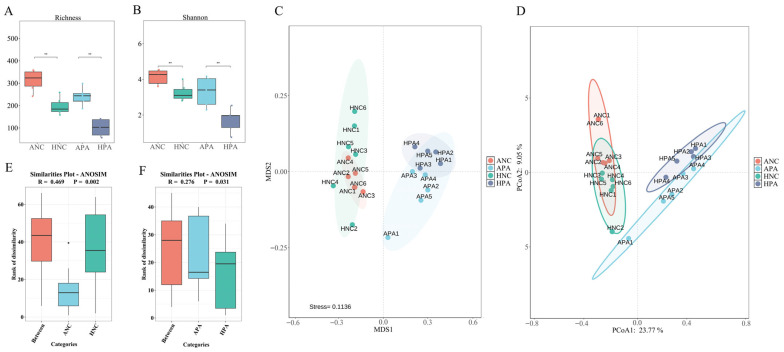
Alpha and beta analyses. Wilcoxon test of Richness (**A**) and Shannon (**B**) indices between same species from two regions (**, *p* < 0.01). Principal coordinates analysis (**C**) and Non-metric multidimensional scaling (**D**) of gut microbiome composition. Each point represents a sequencing sample. Analysis of similarities analysis between same species from two regions ((**E**), ANC vs. HNC; (**F**), APA vs. HPA). ANC: *N. confucianus* in Anhui Province; HNC: *N. confucianus* in Hubei Province; APA: *A. agrarius* in Anhui Province; HPA: *A. agrarius* in Hubei Province.

The Richness and Shannon indices indicated that regional identity had a significant effect on the gut microbiome at the ASV level in both Anhui and Hubei Province (Richness, ANC vs. HNC, *p* ≤ 0.01, APA vs. HPA, *p* ≤ 0.01; Shannon, ANC vs. HNC, *p* ≤ 0.01, APA vs. HPA, *p* ≤ 0.01; Figure 3A,B). The same species exhibited different patterns in these two regions, with significantly greater alpha diversity observed for ANC and APA from Anhui Province than for HNC and HPA in Hubei Province.

At the ASV level, PCoA (Figure 3C) and non-metric multidimensional scaling (Figure 3D) analyses (Bray–Curtis distance) showed that the gut microbiome of the rodents were clustered by host species. These results indicated that the gut microbiome composition is highly similar between different individuals of the same rodent species. However, the ANOSIM analysis showed that the two rodent species in Anhui were significantly different from those in Hubei (Figure 3E, ANC vs. HNC, R = 0.469, *p* = 0.002; Figure 3F, APA vs. HPA, R = 0.276, *p* = 0.031).

### 3.5. MRMs Analysis

Compared to host factors (host phylogeny, weight, and gender), geographic location, temperature, and altitude exerted a greater influence on the gut microbiome (alpha and beta diversity) of the two rodent species (0 < R^2^ > 0.2, *p* < 0.05, Table 1). However, the correlation coefficient for geographical distance was low (0 < R^2^ < 0.2, *p* < 0.05). A substantial proportion of the variance remains unexplained, potentially due to unassessed variables such as diet and environmental pollutants.

### 3.6. Bacterial Co-Occurrence Network and Community Assemblages

**Figure 4:**
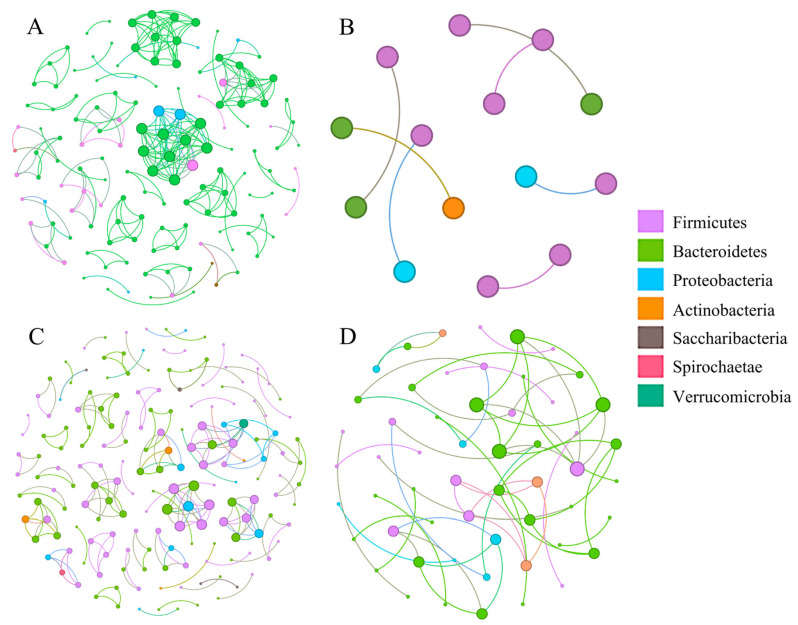
Bacterial co-occurrence network in the APA (**A**), HPA (**B**), ANC (**C**), and HNC (**D**). ANC: *N. confucianus* in Anhui Province; HNC: *N. confucianus* in Hubei Province; APA: *A. agrarius* in Anhui Province; HPA: *A. agrarius* in Hubei Province. Nodes of different colors represent different phyla, and node size indicates the abundance of OTUs.

Based on the ASV level, we constructed bacterial co-occurrence networks for *N. confucianus* and *A. agrarius* in the two regions (Table 2 and Figure 4). The positive proportion of bacterial co-occurrence networks in the two rodent species from the two regions was greater than the negative proportion. The microbial co-occurrence network in the species from Anhui surpassed that from Hubei in terms of nodes (139 APA vs. 14 HPA; 172 ANC vs. 55 HNC), edges (253 APA vs. 7 HPA; 197 ANC vs. 53 HNC), modularity (0.888 APA vs. 0.857 HPA; 0.960 ANC vs. 0.909 HNC), and average degree (3.640 APA vs. 1.000 HPA; 2.291 ANC vs. 1.927 HNC).

**Figure 5:**
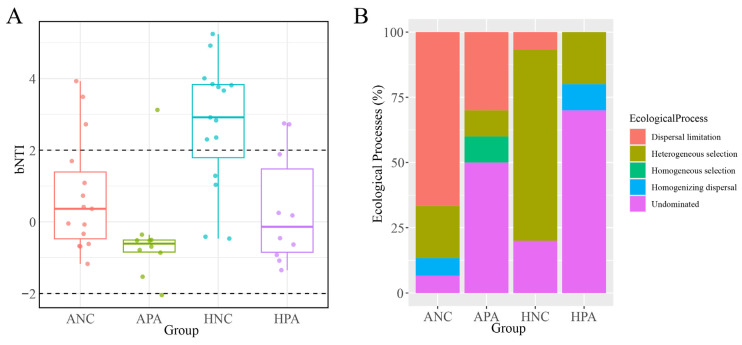
Evaluating the mechanisms of gut microbiome assembly in the same species from two regions using a null model analysis. (**A**). Contribution of deterministic processes (|βNTI| ≥ 2) and stochastic processes (|βNTI| < 2) to gut microbiome assembly of the two rodent species from two regions. (**B**). Proportion of gut microbiome assembly mechanisms in the two rodent species from two regions. ANC: *N. confucianus* in Anhui Province; HNC: *N. confucianus* in Hubei Province; APA: *A. agrarius* in Anhui Province; HPA: *A. agrarius* in Hubei Province.

Using null models, we investigated the assembly processes of the gut microbiome of *N. confucianus* and *A. agrarius* in the two regions. Figure 5A showed that in the HNC, deterministic processes (|βNTI| > 2) generally dominated the assembly of bacterial communities, while in ANC, APA, and HPA, stochastic processes (|βNTI| < 2) dominated. The proportion of dispersal limitation was significantly higher in the gut microbiome of the two rodent species from Anhui region than from Hubei (Figure 5B). The proportion of heterogeneous selection was significantly higher in the gut microbiome of the two rodent species in Hubei region compared to those in Anhui region, especially HNC.

## 4. Discussion

Different geographical regions can significantly alter alpha diversity, beta diversity, bacterial abundance, co-occurrence network characteristics, and assembly mechanisms of the gut microbiome in wild species. Many studies have indicated that habitat environmental factors are the main drivers of alpha and beta diversity in wild animals [1,3,15,26]. In our study, the gut microbiome of the two rodent species from the same region had the same alpha diversity pattern. Through alpha diversity differential analysis, PCoA, and ANOSIM, we found that the gut microbiome composition, alpha and beta diversity of the same species differed significantly across the different regions.

At the network level, the increased edge count and high proportion of positive connections in the Anhui region suggest more frequent and potentially cooperative microbial interactions within the gut communities of both rodent species. Such modular topological patterns have been shown to reflect functional units and potential ecological niches within microbial communities, supporting the interpretation that regional factors foster distinct inter-bacterial associations [27,28,29]. The increase in modularity may reflect a denser functional association and/or niche partitioning in the gut microbiome of the two rodent species in Anhui. The bacterial co-occurrence networks of the two rodent species in Anhui had a higher average degree (3.640 APA vs. 1.000 HPA; 2.291 ANC vs. 1.927 HNC); thus, these species exhibited “small world” characteristics. In “small world” systems, most nodes have more local connections, which allows interaction information (such as metabolites) to rapidly spread throughout the entire microbial community [30,31,32]. The null models revealed that the proportion of dispersal limitations in the gut microbiome of the two rodent species from Anhui region was significantly higher than that in the species from Hubei region. Previous studies have shown that dispersal limitations and deterministic processes are the primary factors promoting bacterial community diversity [33,34]. Furthermore, the gut microbiome of the two rodent species from Anhui was influenced by four assembly processes, whereas that of the rodents from Hubei was influenced by three assembly processes. This suggests that the gut microbiome of the two rodent species from Anhui was affected by more complex dynamics. Therefore, the interactions between the gut microbiome of the two rodent species from Anhui was more complex and may have more intricate dynamics relative to that in the rodents from Hubei [10,35].

However, the proportion of heterogeneous selection in the gut microbiome of the two rodent species from Hubei was significantly higher than that in the rodents from Anhui. This result indicates that selection pressure may have been exacerbated in the two rodent species from Hubei. Increased selection pressure reduces the role of random processes in the gut microbiome assembly. Our MRM analysis showed that geographic regions had limited explanatory power (R^2^ < 0.2), while host phylogeny, gender, and weight exhibited no significant influence. Moreover, the MRM analysis revealed that factors such as elevation and temperature exhibited consistent R^2^ values with geographic location. Therefore, multiple environmental factors—including elevation and temperature across different geographic regions—collectively influenced rodent gut microbiota diversity. This indicated that geographic regional factors should be considered as an integrated unit and not analyzed in isolation [36,37,38]. However, the R^2^ value for geographic location was low (0 < R^2^ < 0.2), indicating that over 80% of the variation was explained by other factors, such as differences in microbial pools, diet, and pollutant exposure. Winter food scarcity occurs in both study regions, with limited residual grains available from Hubei’s farmlands. Although dietary factors are likely influential, we cannot assess their role due to insufficient compositional data. Notably, the sampling sites in Hubei were closer to human settlements and likely experienced higher pollution levels than those in Anhui. Since pollutants can significantly reshape gut microbiota structure and assembly, we suggest that pesticide exposure may be a strong candidate driver of the observed differences.

This hypothesis was further supported by the results of the STAMP analysis. A greater number of beneficial bacteria was significantly enriched in the gut microbiomes of the two rodent species in Anhui, indicating that the gut microbiome composition of rodents in Anhui is more stable. For example, *Pediococcus* is an important genus of lactic acid bacteria that contains a wide range of probiotic bacteria [39,40]. Researchers have found that *Pediococcus* spp. can effectively improve the gut microbiome and inflammatory status of mice [41,42]. Of note, *Petrimonas*, *Ruminiclostridium*, *Alloprevotella*, and *Proteiniphilum* are associated with the generation of short-chain fatty acids [43,44,45,46], which can provide energy for animal life activities and maintain gut homeostasis [47,48].

## 5. Conclusions

We report that the gut microbiome of *N. confucianus* and *A. agrarius* from the same region had similar alpha diversity pattern, co-occurrence network characteristics, and assembly mechanisms. Different geographic regions can affect the gut microbiome composition and co-occurrence networks of the gut microbiome in the same rodent species. For example, the two rodent species from Anhui region exhibited “small world” characteristics and possessed more complex microbial community structures relative to those from Hubei. The assembly processes of the gut microbiome (null model analysis) can partially explain this result. Geographic region exerted a limited yet significant effect (0 < R^2^ < 0.2, *p* < 0.05) on the gut microbiome, surpassing the influence of host phylogeny, gender, and weight in the MRM analysis. However, the influence of regional factors including environmental microbes, pollutants, and food composition, remains to be evaluated. Thus, whether they represent the main drivers of gut microbiota variations in these rodents requires further study.

## Figures and Tables

**Table 1:** MRM results of two rodent species from two different geographical locations.

Factors	ANC/HNC				APA/HPA			
	Alpha Diversity		Beta Diversity		Alpha Diversity		Beta Diversity	
	R^2^	*p*	R^2^	*p*	R^2^	*p*	R^2^	*p*
Weight	0.056	0.054	0.020	0.265	0.008	0.694	0.002	0.835
Gender	0.011	0.343	0.022	0.266	0.004	0.615	0.003	0.702
Geographic location	0.196	0.009	0.166	0.0002	0.154	0.011	0.064	0.021
Altitude	0.196	0.010	0.166	0.001	0.154	0.012	0.064	0.019
Temperature	0.196	0.011	0.166	0.0009	0.154	0.011	0.064	0.017
Phylogenetic branch length	0.029	0.099	0.007	0.534	0.005	0.634	0.043	0.254

**Table 2:** Properties of bacterial co-occurrence network.

Properties	APA	HPA	ANC	HNC
Nodes	139	14	172	55
Edges	253	7	197	53
Modularity	0.888	0.857	0.960	0.909
Network density	0.026	0.077	0.013	0.036
Positive proportions	90.909%	85.714%	97.462%	100%
Negative proportions	9.091%	14.285%	2.538%	0
Average Degree	3.640	1.000	2.291	1.927

Note: ANC: *N. confucianus* in Anhui Province; HNC: *N. confucianus* in Hubei Province; APA: *A. agrarius* in Anhui Province; HPA: *A. agrarius* in Hubei Province.

## Data Availability

The 16S rRNA gene sequencing data are available at the Genome Sequence Archive in BIG Data Center (Accession number PRJCA043574) and *CYTB* sequences are available at the ScienceDB (https://doi.org/10.57760/sciencedb.29591).

## Supplementary Materials

The following supporting information can be downloaded at: https://www.mdpi.com/article/10.3390/ani15213187/s1, Table S1: Detailed information of specimen in this study; Table S2: Species identification information; Figure S1: Phylogenetic tree of two rodent species based on *CYTB* gene.
