# Supplementary material for: Gut Microbiome of Two Rodent Species (Niviventer confucianus and Apodemus agrarius) from Two Regions Exhibit Different Structures and Assembly Mechanisms
Source: Animals (Basel). 2025 Nov 1;15(21):3187. doi: 10.3390/ani15213187 (PMC12609359; doi:10.3390/ani15213187)
Supplement: Supplementary file 1 [file animals-15-03187-s001.zip › Table S2.pdf]

Table S2. Species identification information.

Sequencing number	Query coverage (%)	Percent identity (%)	Species	Compared to mitochondrion accession (NCBI)
**S4300**	100	100	*N. confucianus*	MZ561224.1
**S4310**	100	100	*N. confucianus*	MZ561224.1
**S4327**	100	99.91	*N. confucianus*	MZ561224.1
**S4328**	100	100	*N. confucianus*	MZ561224.1
**S4329**	100	100	*N. confucianus*	MZ561224.1
**S4330**	100	100	*N. confucianus*	MZ561224.1
**S4302**	100	100	*A. agrarius*	MT113512.1
**S4304**	100	99.47	*A. agrarius*	MT113512.1
**S4312**	100	99.47	*A. agrarius*	MT113512.1
**S4315**	100	99.74	*A. agrarius*	MT113512.1
**S4317**	100	99.74	*A. agrarius*	MT113512.1
**S4491**	100	100	*N. confucianus*	MZ561224.1
**S4495**	100	99.65	*N. confucianus*	MZ561224.1
**S4496**	100	100	*N. confucianus*	MZ561224.1
**S4501**	99	100	*N. confucianus*	MZ561224.1
**S4502**	100	100	*N. confucianus*	MZ561224.1
**S4488**	100	100	*N. confucianus*	MZ561224.1
**S4489**	NA	NA	*A. agrarius*	MT113512.1
**S4490**	100	99.39	*A. agrarius*	MT113512.1
**S4492**	100	99.56	*A. agrarius*	MT113512.1
**S4494**	100	99.47	*A. agrarius*	MT113512.1
**S4498**	100	99.74	*A. agrarius*	MT113512.1

Note: ANC: *N. confucianus* in Anhui Province; HNC: *N. confucianus* in Hubei Province; APA: *A. agrarius* in Anhui Province; HPA: *A. agrarius* in Hubei Province.
