# Supplementary figures and images for: Gut Microbiome of Two Rodent Species (Niviventer confucianus and Apodemus agrarius) from Two Regions Exhibit Different Structures and Assembly Mechanisms
Source: Animals (Basel). 2025 Nov 1;15(21):3187. doi: 10.3390/ani15213187 (PMC12609359; doi:10.3390/ani15213187)

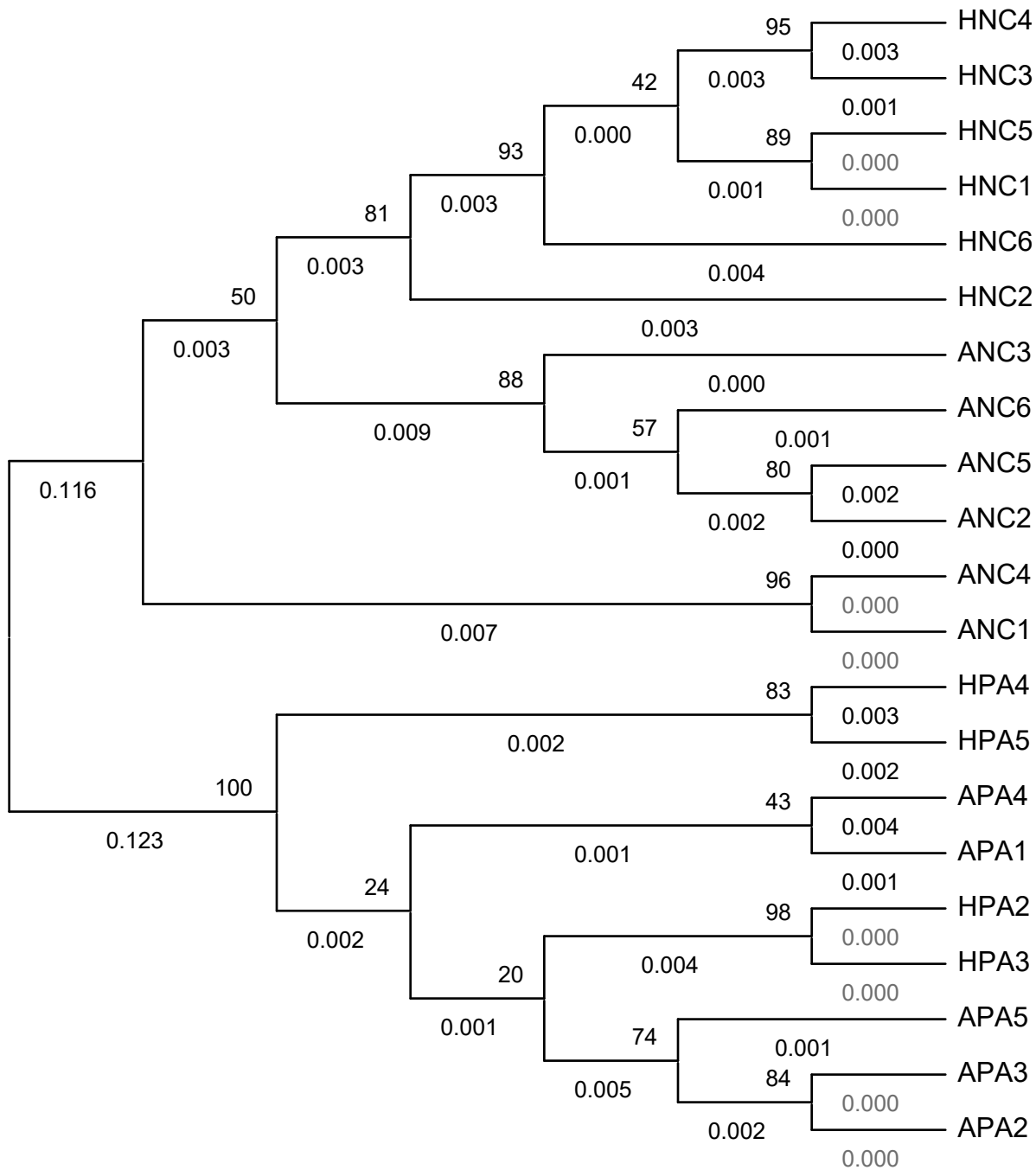

Supplement: Supplementary file 1 [file animals-15-03187-s001.zip › Figure S1.pdf]
